# Challenges and Solutions in Clinical Workflow for the Rehabilitation of Completely Edentulous Patients Through CAD/CAM Dentures: A Case Study

**DOI:** 10.7759/cureus.55394

**Published:** 2024-03-02

**Authors:** P S Manoharan, Priyasha R Wase, Sneha Sivakumar

**Affiliations:** 1 Department of Prosthodontics and Crown & Bridge, Indira Gandhi Institute of Dental Sciences, Pondicherry, IND

**Keywords:** dental education, digital denture, completely edentulous patients, removable denture, cad cam

## Abstract

In the field of removable prosthodontics, computer-aided design and computer-aided manufacturing (CAD/CAM) have become widely recognized. The traditional method, which uses heat-polymerized resins for injection or compression molding, necessitates up to five patient visits and laborious laboratory processes. A digital workflow combined with a CAD/CAM methodology can provide prompt prosthesis delivery for patients with time constraints. This article's goal is to outline the steps and the limitations in the fabrication of digital dentures as well as the challenges, limitations, and solutions developed while developing a clinical workflow for the rehabilitation of completely edentulous patients with the CAD/CAM System.

## Introduction

Traditional methods of fabricating complete dentures (CD) include multiple stages and employ multiple materials and techniques, running the risk of introducing human errors at every stage; this could result in a possible loss of accuracy of the denture in the final stage [1]. Digital technology in denture fabrication claims that such errors can be minimized as the number of appointments are reduced. Direct scanning of the impressions and jaw relations is possible, which reduces errors in the usage of multiple materials and techniques. Computer-aided design and computer-aided manufacturing (CAD/CAM) technology have revolutionized many fields of dentistry practiced in dental offices as well as dental laboratories, such as the fabrication of inlays, onlays, fixed partial dentures, and maxillofacial prostheses. With the recent developments, different commercially available systems have been developed for the fabrication of complete dentures [2]. At present several systems are available for the fabrication of digital dentures: AvaDent (Global Dental Science, Scottsdale, AZ); Ceramill Full Denture System (Amann Girrbach AG, Koblach, Austria); Baltic Denture System® (Merz Dental GmbH, Lütjenburg, Germany); DENTCA/Whole You (DENTCA, Inc.,Torrance, CA; Whole You, Inc, Brooklyn, NY); Wieland Digital Denture (Ivoclar Vivadent, Inc., Schaan, Liechtenstein); Vita Vionic (VITA Zahnfabrik, Bad Säckingen, Germany) [3].

The ongoing objective is to make it easier to quickly and affordably fabricate computer-engineered complete dentures (CECD) with acceptable success factors. Numerous studies have been published in the previous few decades demonstrating the significant amount of interest this topic has attracted [4-7]. Typically, digital designing and data (records) scanning are used to create CECDs after which the denture is then milled using computerized numerical control.

Most digital denture systems claim to provide dentures in two appointments [8-10]. Past research and the evidence from the literature state that there are a lot of areas that are crucial and need expertise and understanding; however these are not conventionally covered in regular training programs. To explore more into the relatively unknown areas pertaining to this topic, this article is presented as a case study to explore the details of the clinical workflow in digital denture fabrication. 

## Case presentation

The present study was planned in order to validate the superiority claims proposed by digital dentures. The system used in the case study for fabrication of digital dentures was Baltic Denture System® (BDS). This system has a set of exclusive instruments and materials to be used for fabrication which are listed in Table 1. For making the edentulous impression and jaw relation, monomer-free thermoplastic resin was used along with the components of the BDS system.

**Table 1:** Components of the Digital Denture System * Baltic Denture System®, Merz Dental GmbH, Lütjenburg, Germany [11]

Components of the Digital Denture System*	Composition	Characteristics/ Features
Upper KEY / Lower KEY	Copolymer based on polymethylmethacrylate (PMMA)	Used for building up of the functional ridge
BD KEY^®^ Lock	Copolymer based on polymethylmethacrylate (PMMA)	Used for connecting the upper and the lower BD KEY^®^
BD KEY^® ^Plane / BD KEY^®^ Fin / BD KEY^®^ Connect	Polyetheretherketone (PEEK), Carbon fiber-reinforced	Alignment of the interpupillary line and ala- tragal line.

A 55-year-old male patient reported to the department of prosthodontics in our institution with the chief complaint of ill-fitting complete dentures. The patient was a denture wearer for the last five years. Intraoral examination and evaluation of the dentures revealed a moderately resorbed mandibular ridge and severely attrited and ill-fitting dentures. With informed consent obtained from the patient, two types of dentures, conventional and digital dentures were planned. There were no additional appointments required for the patient apart from those that were used for the fabrication of the conventional dentures as well. The expected clinical outcomes, patient-reported outcome measures through structured feedback, and operator perception was recorded after wearing each of the dentures separately. The patient was blinded during the denture delivery stage. 

**Figure 1:**
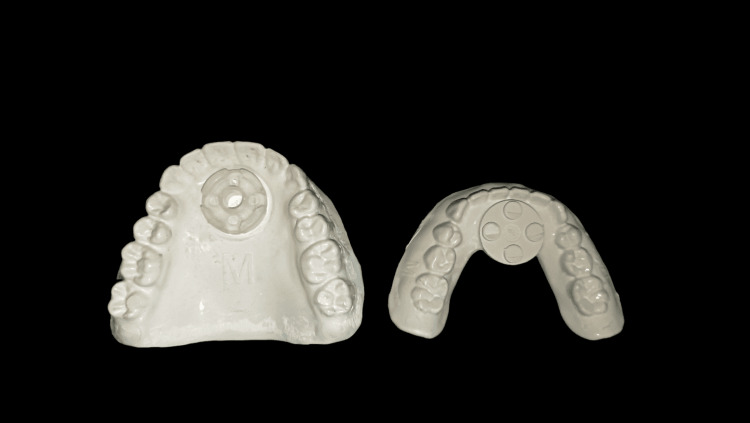
BD Keys BD: Baltic Denture

Careful selection of the patient was made with the evaluation of the maxillary and mandibular bone height, ridge morphology, inter-arch space, and the maxilla-mandibular relationship (Class I). Selection of the Baltic Denture (BD) Key was done intraorally taking into consideration the patient's oral anatomical features (Figure 1). 

The vertical dimension at rest and vertical dimension at occlusion were determined and marked on a wooden tongue depressor prior to the start of the case. These keys are available in three sizes (small, medium, and large). The BD Key was selected for the size closest to the maxillary arch; the BD Plane and the BD Fin were assembled together and connected to the selected BD Key.

**Figure 2:**
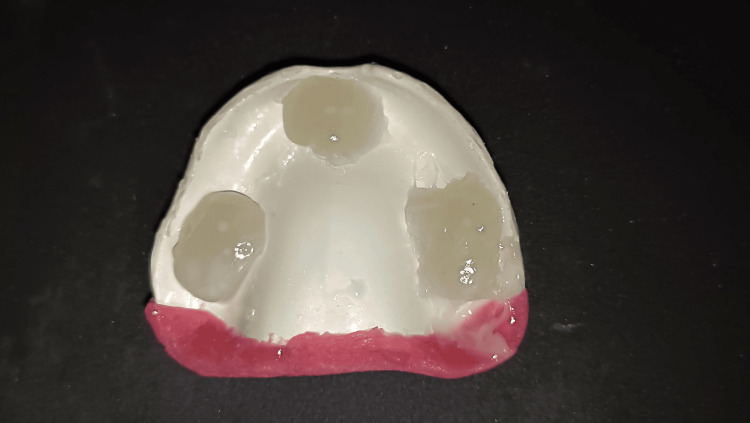
Maxillary BD Key with three stops placed BD: Baltic Denture

**Figure 3:**
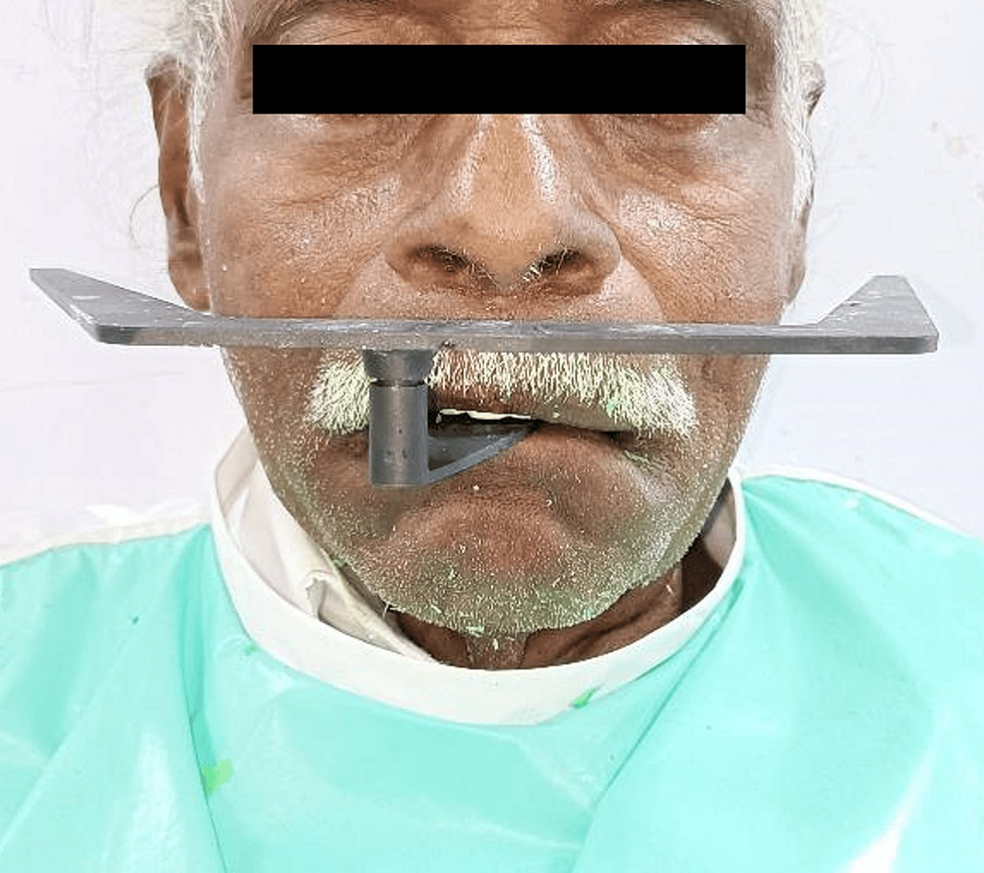
BD Plate and BD Fin attached to the BD Lock in the BD Keys BD: Baltic Denture

The pellets of the thermoplastic resin were softened in a water bath (hot water) at 75-85°C which was hardened to a plastic compound at the oral temperature. Three basal stops with the resin were placed on the upper key, and then the assembly of the BD Key, BD Plane, and BD Fin were held together in the patient’s mouth until it was set (Figure 2). While the stops were curing, the teeth of the upper key were aligned according to the functional aspects i.e., Camper's plane and inter-pupillary line. Aesthetic considerations i.e., the center of the face, length of the anterior teeth, and the smile line were aligned by holding the key in place until the resin was set (Figure 3).

**Figure 4:**
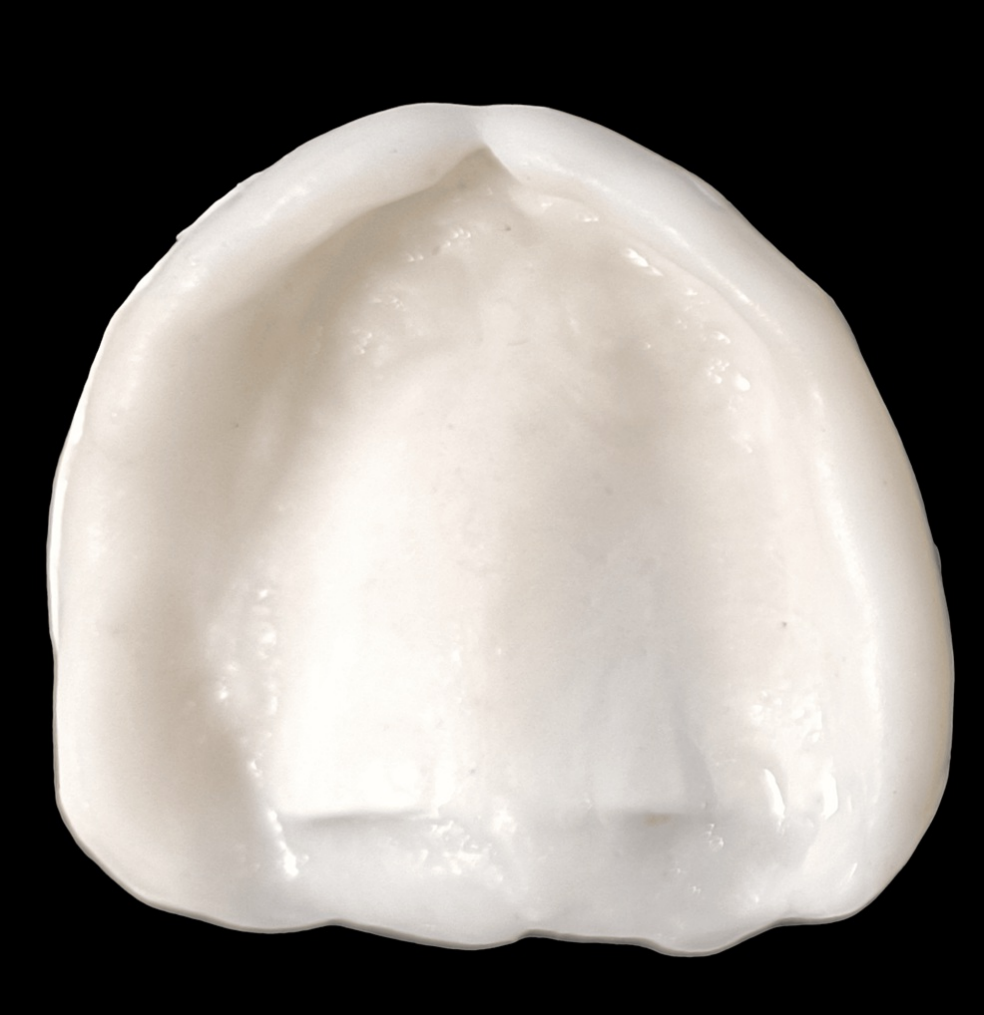
Functional build-up of the maxillary ridge

**Figure 5:**
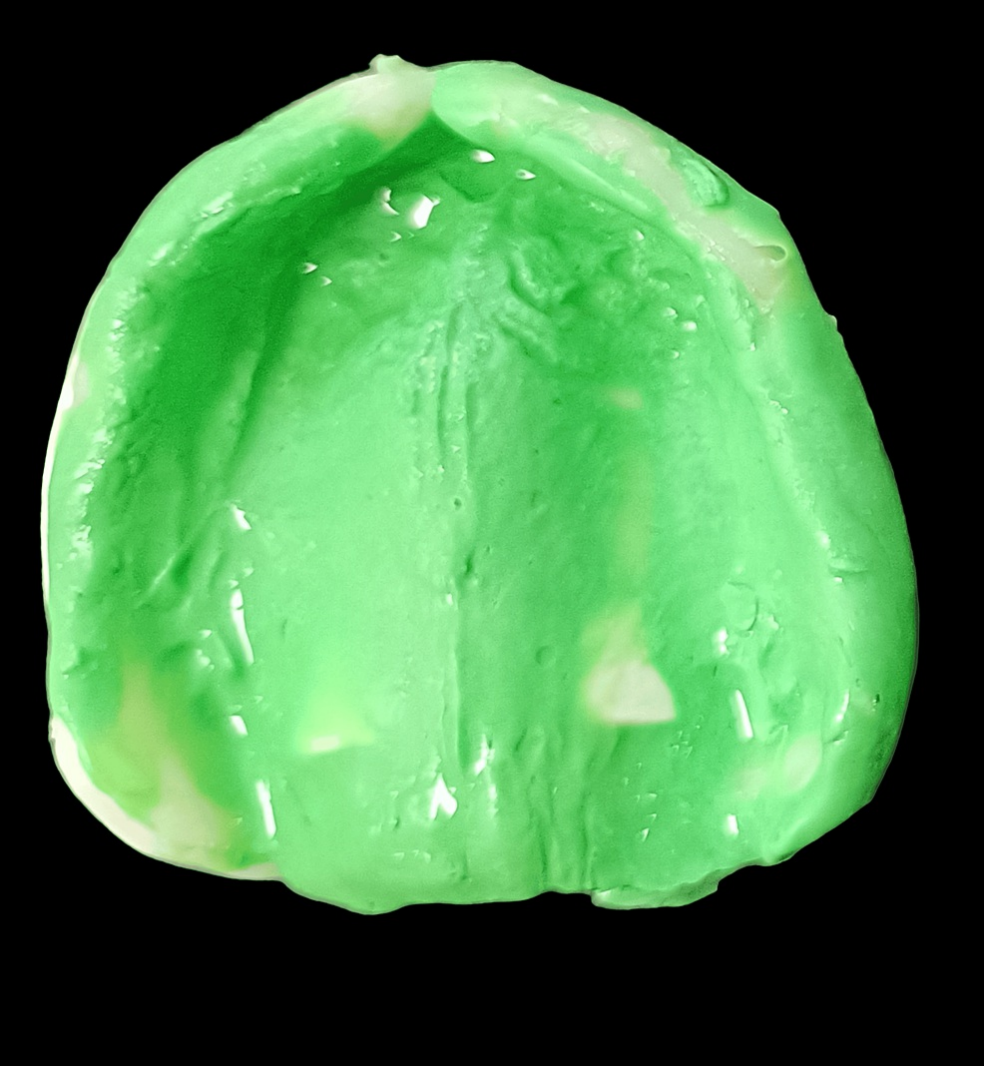
Functional impression of the maxillary ridge

By further addition of the resin, the functional ridge and the vestibules were built up (Figure 4). Functional impressions were made using the prepared upper key with light-body impression material and were shaped under functional movement (Figure 5).

**Figure 6:**
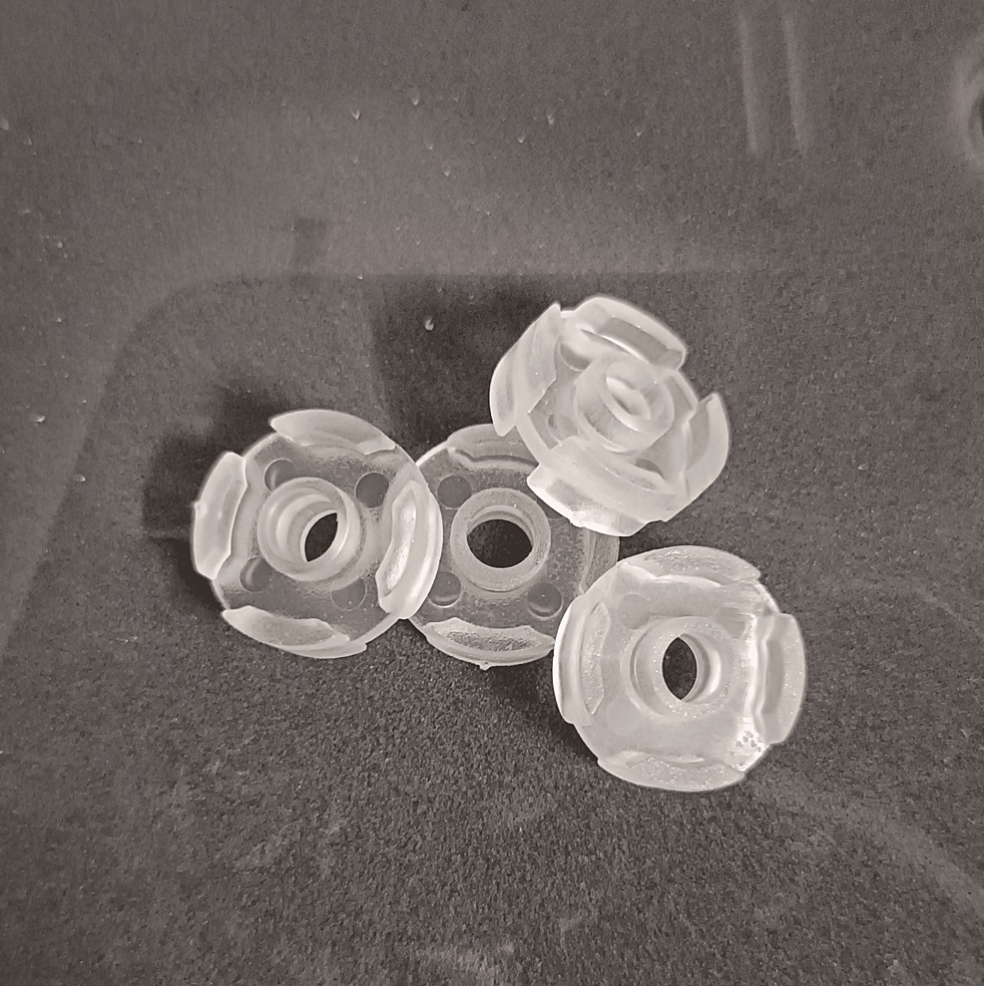
BD Key Lock BD: Baltic Denture

The upper key and lower key were connected using the BD key lock (Figure 6). The spatial conditions between the alveolar ridge and the intaglio surface of the lower key were checked by locking the upper and lower key.

**Figure 7:**
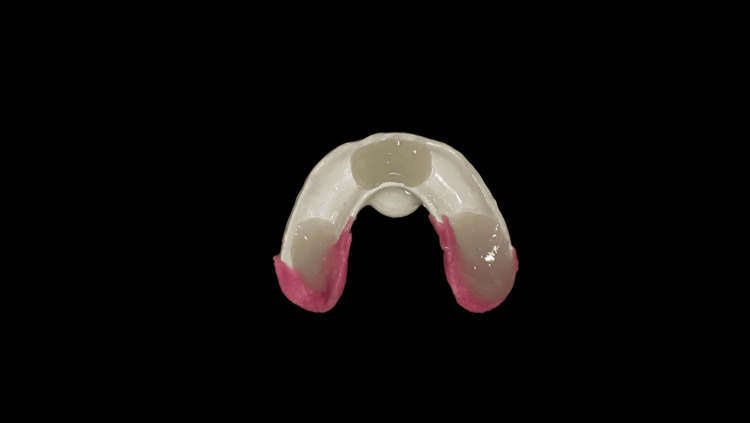
Three stops placed on the mandibular key

**Figure 8:**
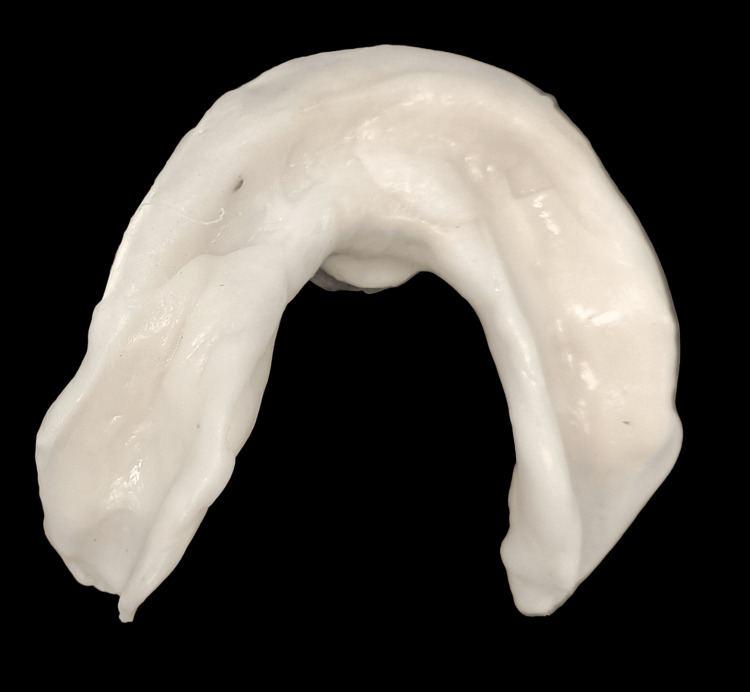
Build-up of the mandibular key

**Figure 9:**
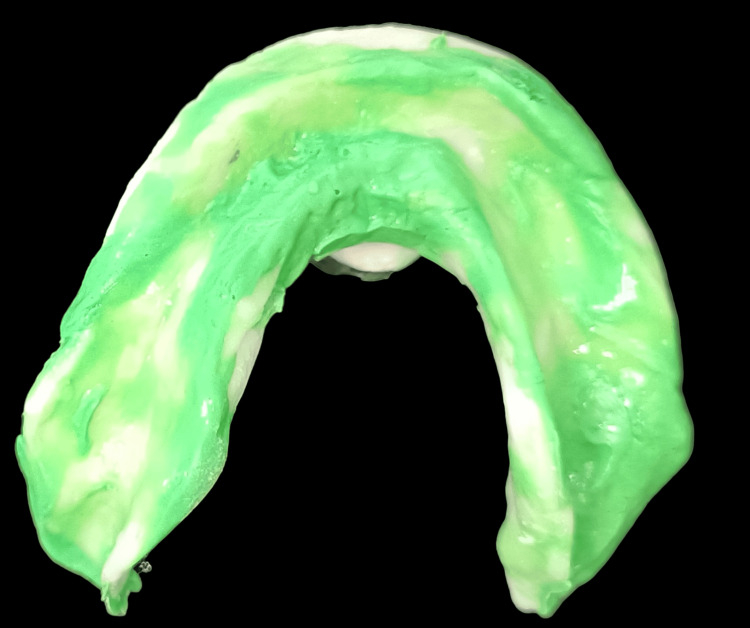
Functional impression of the mandibular key

Similar to the upper key, three stops were placed in the basal area of the lower key with the resin (Figure 7). The patient was guided into his centric terminal occlusion. The vertical and horizontal relationship between the upper and lower jaws were determined and verified. Then the lower vestibular ridge was built up with the resin and was shaped under functional movement in the mouth. The two keys were then separated and the lingual functional ridge was built up using tongue movements (Figure 8). A final check of the maxillomandibular relationship (centric position and vertical dimension with the record marked on the tongue depressor) was carried out before making the functional impression (Figure 9).

The earlier procedures to determine the relationship between the vertical and horizontal positions of the upper and lower jaws were repeated and adjusted in the event that there were any notable variations. The upper key and lower key were kept interlocked, and the entire surface of the lower key was filled with light-body impression material for making impressions with functional movement.

**Figure 10:**
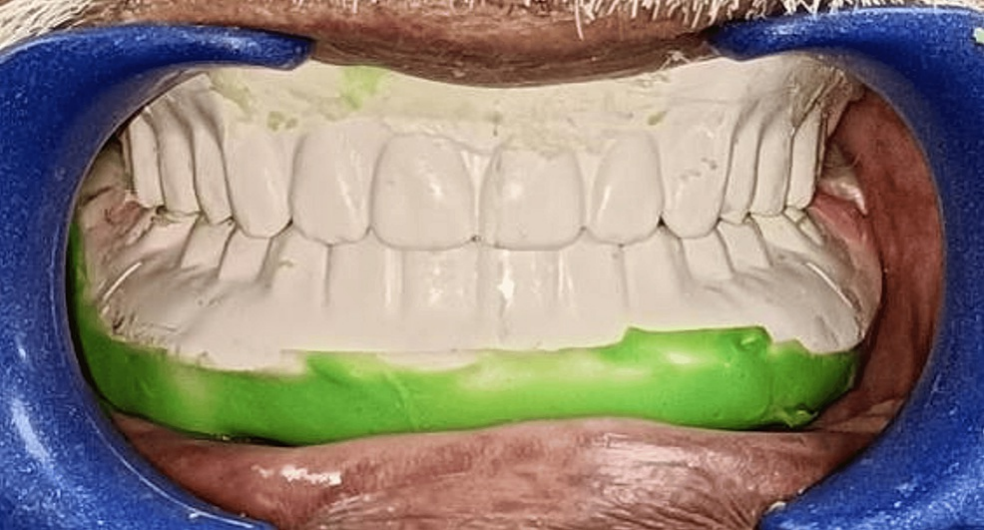
Locked maxillary and mandibular key

With the midline marked, photographs were taken in the frontal and lateral view, with the lips in the rest position and in the smiling position to assist the dental laboratory technician in designing the teeth arrangement as well as fabrication of the dentures (Figure 10). Records and impressions were sent to the dental laboratory when the patient was satisfied and the functionality and aesthetics were re-evaluated, in addition to detailed guidelines for teeth arrangements and a full-face image for designing the smile.

**Figure 11:**
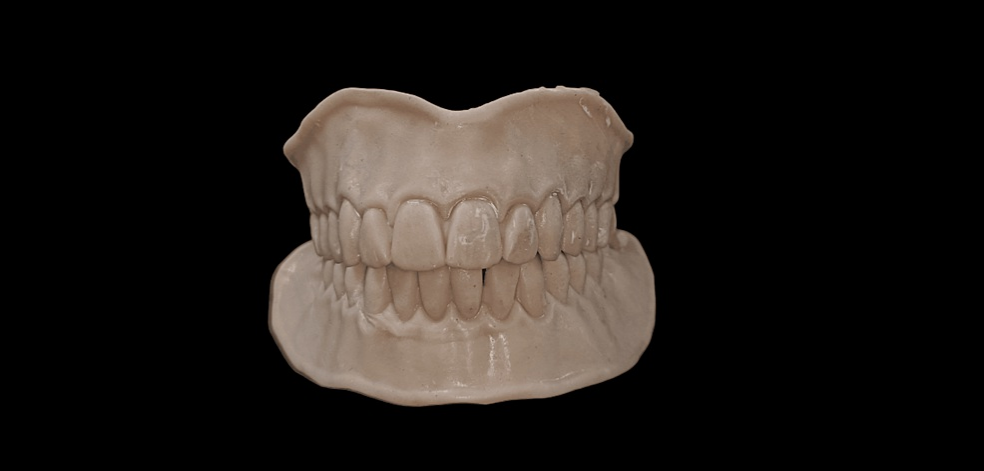
Trial denture

After scanning the records, a digital tooth arrangement was completed. A prototype/trial denture was produced using the STL file made from this design (Figure 11). The conventional wax tooth try-in was replaced by the trial denture, which allowed for the same clinical evaluations of aesthetics, phonetics, occlusion, vertical dimension, and other factors. As the printed try-in was intended to duplicate the final denture base, the denture flange and extensions, fit, and retention of the denture base could also be assessed (as with a processed record base). Any alterations that required either additive or subtractive work were done in the trial denture before being sent to the lab for final finishing touches. The dentures can be milled in the BD Load or conventionally by subtractive technique. The difficulties encountered during the procedural steps are mentioned in Table 2. 

**Table 2:** Problems and their possible causes, as well as the solutions

Problems and possible reasons	Solutions
Over-extended / underextended trays: pre-formed trays proved a problem during recording the borders accurately.	Select one size lower tray and modify the extensions with self-cure acrylic resin, which can be later removed.
Retroclined anterior teeth affecting the aesthetics: design and virtual teeth arrangement issues	Anterior teeth are built up and are contoured with self-cure acrylic/composite material
Increased vertical dimension: scanning errors / key lock dislodged	Maxillary trial denture checked for visibility and plane shift. Vertical dimension at rest and occlusion was measured. Teeth from the trial denture were trimmed to reduce the vertical dimension and the plane was corrected. Wash impressions were made for both upper and lower dentures. Bite registration was done with thermoplastic material.
Midline deviation: design and virtual teeth arrangement	New midline was marked in the trial denture
Occlusion discrepancy was observed outside the patient’s mouth as well as inside the patient’s mouth	Selective grinding was done

**Figure 12:**
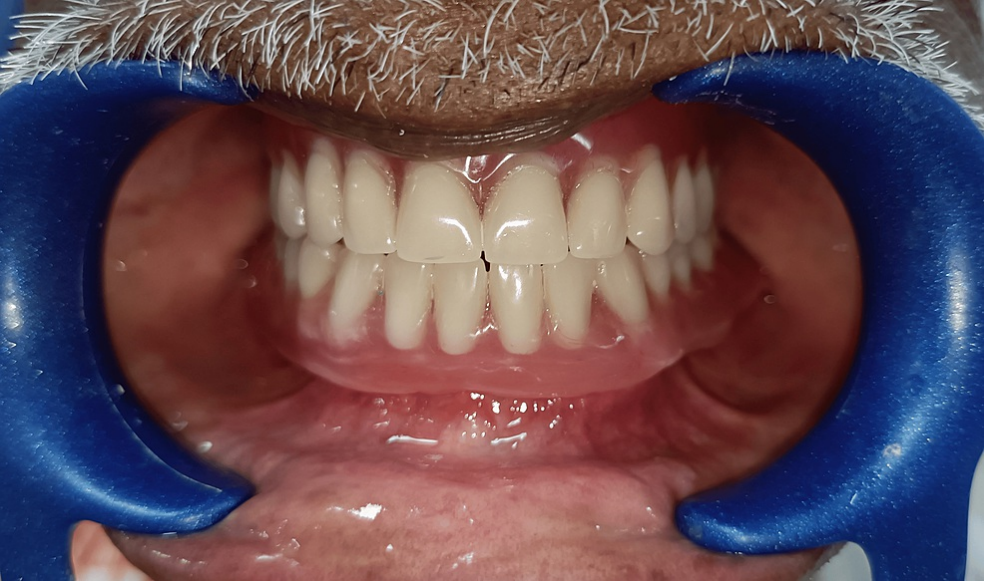
Conventional denture

**Figure 13:**
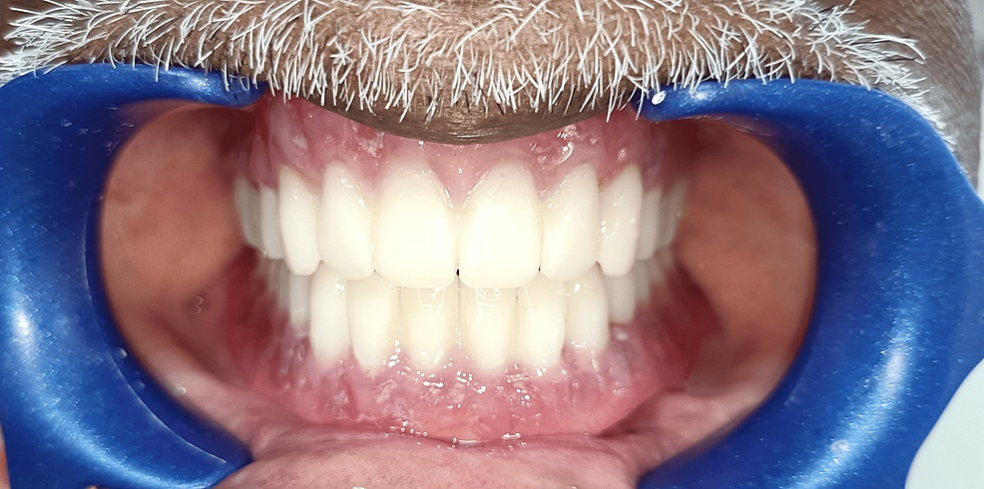
CAD/CAM denture CAD/CAM: computer-aided design and computer-aided manufacturing

One set of conventional complete dentures was also fabricated within the five-appointment process, namely: (1) preliminary impressions; (2) border molding and final impressions; (3) interocclusal records and tooth selection; (4) teeth wax trial placement; and (5) denture adjustment. The conventional complete dentures (CCDs) were fabricated with the conventional lost wax technique using heat-polymerizing acrylic resin (Figure 12). After the fabrication of the digital denture, it was checked for adaptation, occlusion, and aesthetics in the patient's mouth (Figure 13).

## Discussion

Encouraging advances in computer-aided technology in the field of CD fabrication have increased the interest and the number of publications describing the same in the last few years [9,12,13]. Instead of conventional prostheses, this study aims to provide more evidence supporting the clinical usage of digital prostheses.

It has been observed that CAD/CAM dentures have less resin volume and weight than conventional dentures, which can improve patient comfort and adaptability [10,12-14]. Research indicates that the fit of CAD/CAM dentures is superior to that of traditional ones, hence improving patient's acceptance and tolerance [15-17]. Advantages include shorter treatment durations, fewer clinical visits, and less discomfort for patients. The advantages of the material used include decreased porosity, which prevents the growth of bacteria; and decreased polymerization shrinkage, which leads to good adaptation [15,16].

The Baltic Denture System's greatest benefit is that it requires fewer clinical visits, which is advantageous to patients and clinicians, due to the rows of teeth that are attached to the BD Keys for both the patient and the clinician, along with a visual of the final denture in the first appointment itself. The denture delivery is ensured at the second visit in comparison with other CAD/CAM systems currently available and is referred to as well as compared with the other materials that are available in the market [18-19]. The challenges faced and limitations observed during the process of fabrication of the denture with this system as well as solutions are listed in Table 2.

One of the limitations this system possesses is the inability to precisely evaluate the centric relation as the keys are prefabricated BD keys that can only be locked into a Class I relationship, which doesn’t allow freedom of movement for Class II and Class III ridge relationships and is only applicable to individuals with a good Class I maxillomandibular connection. In addition, establishing the mandibular occlusal plane is not possible. It was difficult to assess the lip support, maxillary incisal edge position, buccal fullness, and the mandibular occlusal plane were challenging with the preformed keys provided, because the keys might be under-extended or over-extended which gives a false result, due to over-extension of the trays into the tissues. Fabrication of a trial denture helped overcome the aforementioned problems. Also, the current material and laboratory costs are higher than those of the traditional methods. The patient reported better aesthetics with the conventional denture when compared with the CAD/CAM dentures but commended the good fit and retention with the CAD/CAM dentures.

## Conclusions

Many studies have been done comparing the CCEDs and CCDs, however, they have not mentioned the drawbacks pertaining to the digital workflow and the fabrication process. One of the biggest shortcomings is the inability to precisely verify the centric relation and digitally customize the dentures. Another drawback, when compared to conventional techniques, is that at present keys are beneficial only for patients with a favorable Class I maxillomandibular relationship. However, there is a need for further investigation supported by long-term clinical studies as CAD/CAM dentures are a relatively new technology that will likely not go away.

## References

[1] Jacob RF (1998). The traditional therapeutic paradigm: complete denture therapy. J Prosthet Dent.

[2] Masih A, Choukse V, Srivastava R, Sharma N (2018). Review CAD CAM complete dentures: a review. MP State Dental Journal.

[3] Steinmassl PA, Klaunzer F, Steinmassl O, Dumfahrt H, Grunert I (2017). Evaluation of currently available CAD/CAM denture systems. Int J Prosthodont.

[4] Andreescu CF, Ghergic DL, Botoaca O, Hancu V, Banateanu AM, Patroi DN (2018). Evaluation of different materials used for fabrication of complete digital denture. Mater Plast.

[5] McLaughlin JB, Ramos V Jr, Dickinson DP (2019). Comparison of fit of dentures fabricated by traditional techniques versus CAD/CAM technology. J Prosthodont.

[6] Lo Russo L, Salamini A (2018). Removable complete digital dentures: A workflow that integrates open technologies. J Prosthet Dent.

[7] Steinmassl PA, Wiedemair V, Huck C, Klaunzer F, Steinmassl O, Grunert I, Dumfahrt H (2017). Do CAD/CAM dentures really release less monomer than conventional dentures?. Clin Oral Investig.

[8] Steinmassl O, Dumfahrt H, Grunert I, Steinmassl PA (2018). CAD/CAM produces dentures with improved fit. Clin Oral Investig.

[9] Goodacre BJ, Goodacre CJ, Baba NZ, Kattadiyil MT (2016). Comparison of denture base adaptation between CAD-CAM and conventional fabrication techniques. J Prosthet Dent.

[10] Bidra AS, Taylor TD, Agar JR (2013). Computer-aided technology for fabricating complete dentures: systematic review of historical background, current status, and future perspectives. J Prosthet Dent.

[11] (2023). Innovation | Baltic Denture System. Baltic‑Denture‑System.de. https://www.merz-dental.de/en/digital-solutions/digital-denture/bdkeyr-set.

[12] Kattadiyil MT, Jekki R, Goodacre CJ, Baba NZ (2015). Comparison of treatment outcomes in digital and conventional complete removable dental prosthesis fabrications in a predoctoral setting. J Prosthet Dent.

[13] Kattadiyil MT, Goodacre CJ, Baba NZ (2013). CAD/CAM complete dentures: a review of two commercial fabrication systems. J Calif Dent Assoc.

[14] Tew IM, Soo SY, Pow EH (2023). Digitally versus conventionally fabricated complete dentures: A systematic review on cost-efficiency analysis and patient-reported outcome measures (PROMs). J Prosthet Dent.

[15] Deng K, Wang Y, Zhou Y, Sun Y (2023). Comparison of treatment outcomes and time efficiency between a digital complete denture and conventional complete denture: A pilot study. J Am Dent Assoc.

[16] Thu KM, Molinero-Mourelle P, Yeung AW, Abou-Ayash S, Lam WY (2023). Which clinical and laboratory procedures should be used to fabricate digital complete dentures? A systematic review. J Prosthet Dent.

[17] Zissis A, Yannikakis S, Polyzois G, Harrison A (2008). A long term study on residual monomer release from denture materials. Eur J Prosthodont Restor Dent.

[18] Goodacre CJ, Garbacea A, Naylor WP, Daher T, Marchack CB, Lowry J (2012). CAD/CAM fabricated complete dentures: concepts and clinical methods of obtaining required morphological data. J Prosthet Dent.

[19] Schweiger J, Güth JF, Edelhoff D, Stumbaum J (2017). Virtual evaluation for CAD-CAM-fabricated complete dentures. J Prosthet Dent.

